# Vesicular Transport of Progeny Parvovirus Particles through ER and Golgi Regulates Maturation and Cytolysis

**DOI:** 10.1371/journal.ppat.1003605

**Published:** 2013-09-19

**Authors:** Séverine Bär, Jean Rommelaere, Jürg P. F. Nüesch

**Affiliations:** German Cancer Consortium (DKTK), Program “Infection and Cancer,” Division Tumor Virology (F010), German Cancer Research Center (DKFZ), Heidelberg, Germany; Salk Institute, United States of America

## Abstract

Progeny particles of non-enveloped lytic parvoviruses were previously shown to be actively transported to the cell periphery through vesicles in a gelsolin-dependent manner. This process involves rearrangement and destruction of actin filaments, while microtubules become protected throughout the infection. Here the focus is on the intracellular egress pathway, as well as its impact on the properties and release of progeny virions. By colocalization with cellular marker proteins and specific modulation of the pathways through over-expression of variant effector genes transduced by recombinant adeno-associated virus vectors, we show that progeny PV particles become engulfed into COPII-vesicles in the endoplasmic reticulum (ER) and are transported through the Golgi to the plasma membrane. Besides known factors like sar1, sec24, rab1, the ERM family proteins, radixin and moesin play (an) essential role(s) in the formation/loading and targeting of virus-containing COPII-vesicles. These proteins also contribute to the transport through ER and Golgi of the well described analogue of cellular proteins, the secreted Gaussia luciferase in absence of virus infection. It is therefore likely that radixin and moesin also serve for a more general function in cellular exocytosis. Finally, parvovirus egress via ER and Golgi appears to be necessary for virions to gain full infectivity through post-assembly modifications (e.g. phosphorylation). While not being absolutely required for cytolysis and progeny virus release, vesicular transport of parvoviruses through ER and Golgi significantly accelerates these processes pointing to a regulatory role of this transport pathway.

## Author Summary

Previously, it was thought that non-enveloped lytic parvoviruses were released through a lytic burst of cells at the end of infection. However, recent work demonstrated that these small non-enveloped single-stranded DNA viruses are actively transported through vesicles from the nucleus, the site of replication and assembly, to the cell periphery. The current investigation demonstrates that progeny particles become engulfed into COPII-vesicles in the endoplasmic reticulum (ER) and are transported through the Golgi to the plasma membrane (PM). ERM family proteins radixin and moesin appear to play an essential role in this cellular secretion pathway. While passing through ER and Golgi cisternae, PVs maturate through post-assembly modifications, which significantly increase the infectivity of progeny virions. Finally, the vesicular transport of parvoviral particles was shown to regulate virus-induced cytolysis, thereby accelerating the further release and spread of progeny virions. As rodent PVs are currently viewed as oncolytic agents for cancer virotherapy, it is important to further investigate the mechanism of PV egress — not only to improve the spreading of these agents through the tumor mass, but also to optimize the induction of an anti-tumor immune response upon virus — induced cytolysis.

## Introduction

Egress of enveloped viruses is typically associated with the cell secretory pathway guiding the particles and/or their precursors through cellular organelles, in particular the endoplasmic reticulum (ER) and the Golgi cisternae [1]–[7]. In this regard, enveloped viruses usurp the cellular secretory machinery in order to achieve the efficient transport of progeny viruses to the plasma membrane and the maturation of precursor particles into infectious virions [8]–[14]. In contrast, non-enveloped viruses are thought to be released as mature virions through a cytolysis burst at the end of infection [15]–[17]. However, there is evidence on non-enveloped virus egress through active transport besides the major lytic pathway. A few non-enveloped lytic viruses were shown to be actively transported to the cell periphery and released prior to cell lysis. For instance SV40 was detected in intra-cytoplasmic smooth membrane vesicles and described as being released from the cell before cytopathic effects are seen [18]. Similarly, cocksackie B3 virus was found to be transferred from cell to cell through microvesicles [19]. We also recently reported that in the case of non-enveloped lytic parvoviruses that progeny virions are actively transported from the nucleus to the plasma membrane (PM) through vesicles in a gelsolin-dependent manner [20].

The cellular secretory pathway has been characterized in great detail and a number of proteins involved in the specific recognition of cargos, the formation and loading of vesicles, and the guided transport through the cytoplasm have been identified [21]–[28]. Once in the ER, transport of cargos is mediated by coat protein complex II (COPII) vesicles. Engulfment of cargos into COPII vesicles at ER exit sites is triggered by the activation of the small GTPase Sar1, a process which in turn induces the recruitment of two heterodimeric complexes, Sec23–Sec24 and Sec13–Sec31, and their assembly into a protein coat. In this complex, sec23–sec24 is responsible for the selection and binding of the cargo. Sec24 can either bind directly to the transported protein if it features a transmembrane domain, or interact with a transmembrane receptor specific to the cargo in the case of a secreted protein. In both cases, sec23–sec24 bound to the cargo forms a ternary complex which concentrates the cargo and bends membranes. The budding vesicle is then enveloped by the Sec13–sec31 cage and released from the ER by fission.

During the last step of this budding, cellular factors responsible for directing the vesicle to the next compartment and fusing the vesicular membrane with the target membrane, associate with the departing vesicle [29], [30]. Targeting of cargo-vesicles to distinct organelles/compartments through the cytoplasm is achieved by distinct small GTPases, Rab-proteins, which are associated with the surface of moving vesicles. For instance, Rab1 targets vesicles from the ER to the Golgi apparatus, while Rab6 is responsible for transport within the Golgi cisternae and for retrograde transport from the Golgi apparatus to ER. Transport from the transgolgi-network (TGN) to the plasma membrane (PM) can take different routes and is accordingly controlled by a variety of Rab proteins. Among these, Rab8 is responsible for guiding the vesicles along the direct route from the TGN to the PM, while Rab11 shuttles TGN-vesicles through recycling endosomes to the PM [22]. A number of other Rab proteins are also involved in vesicle targeting to the endocytosis and secretion pathways, and new routes are constantly being discovered. When the vesicles reach the periphery of the cell, the release of the cargo is generally achieved by fusion of the vesicular membrane with the PM [31], [32].

Parvoviruses (PV) are small icosahedral non-enveloped particles with a 5.1-kb linear single-stranded DNA genome. During productive infection, PVs induce dramatic morphological and physiological changes in their host cells, culminating in cell death and lysis [17], [33], [34]. This is mainly attributed to the large non-structural viral protein NS1, a multifunctional protein involved in particle production and spread (reviewed in [35]). NS1 was shown to modulate cellular pathways by physical interactions with distinct cell components [36], [37] and/or induction of post-translational modifications of cellular target proteins [3], [20], [37], [38]. These targets might be modified either directly by NS1/CKIIα, a recently described complex formed by NS1 with the catalytic domain of cellular CKII [36], or indirectly through activation/modulation of the PDK1/PKC/PKB signaling cascade [39]. The latter activation is mediated by the virus-induced association of the ERM family protein radixin with PKCη, forming a complex that controls the activity and substrate specificity of the PDK1.

The cellular proteins targeted by the NS1/CKIIα complex include the ERM-family protein radixin [38]. ERM proteins are known mediators of the interplay between filamentous actin and membrane structures [40]. In particular, radixin (Rdx) has been implicated in the parvovirus MVM infectious cycle, controlling progeny particles production and spreading as measured through the formation of lysis plaques [38]. Gelsolin, an actin-severing protein was found to be another target for the NS1/CKIIα complex. Gelsolin shown to contribute to the egress of PV progeny virions [20] through its function in the formation and/or the PV-loading of cellular vesicles, which are then targeted through the cytoplasm to the PM [20]. NS1/CKIIα-mediated phosphorylation seems to play a crucial role in this process.

The present investigation aims to characterize vesicular egress of non-enveloped lytic parvoviruses in more detail and to determine the impact of this process on the PV life-cycle. Using fluorescence microscopy, biochemical fractionation and selective inhibition of proteins involved in vesicular transport (e.g. Rab-proteins), we show that PV progeny particles become engulfed into vesicles in the ER and are actively transported through the Golgi apparatus to the PM. The ERM family proteins moesin and radixin play an important role in the formation/loading and targeting of progeny virus-containing vesicles. Finally, egress through the Golgi apparatus appears to play an essential role in post-assembly modification, infectivity of progeny virions, and the stimulation of virus-induced cytolysis.

## Results

### Progeny PV particles get engulfed into vesicles in the perinuclear area

**Figure 1:**
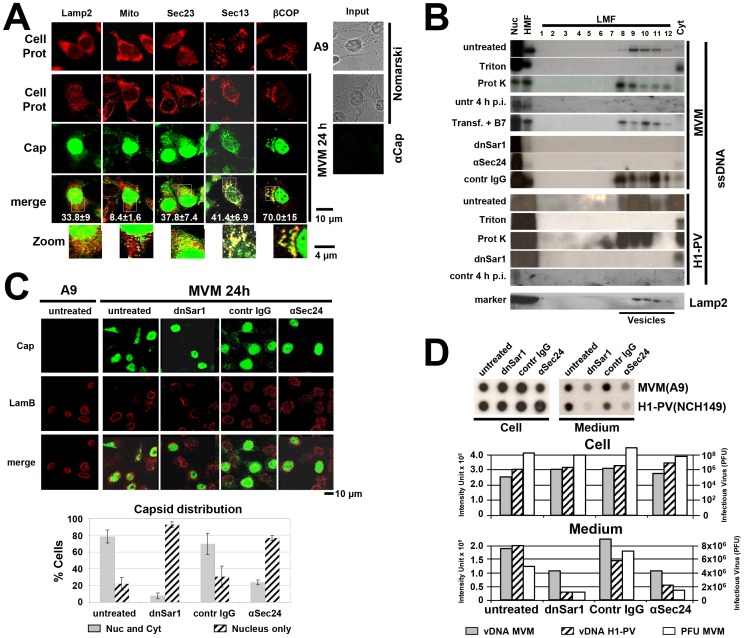
PV progeny particles get engulfed into COPII vesicles. (A) A9 cells grown on spot slides were infected (MVM 24 h) or not (A9) with MVMp (30 pfu/cell), and single rounds of infection were monitored in presence of neutralizing antibody B7 after initial infections. Cells were fixed with paraformaldehyde at 4 h (Input) or 24 h (MVM 24 h) p.i., and analyzed by confocal laser scanning microscopy after double-staining with antisera specific for the indicated cell proteins (red) and MVM capsids (green). Lack of interference from input virions is revealed by the absence of detectable capsid signal at 4 h p.i. (Nomarsky picture). Colocalization areas appear yellow in the merge panel and are expressed as percent of total cytosolic capsids after quantification by Image J analysis of 10 infected cells from three individual experiments. To better visualize colocalization, zoom-ins were performed on indicated areas. Bars indicate scales (in µm). (B–D) A9 cells were infected with MVMp (30 pfu/cell), NCH149 cells with hgH1-PV (30 pfu/cell) and harvested/fixed at 24 h p.i. When indicated, Sar1 functioning was jeopardized by over-expression of the dominant-negative Sar1K38M variant (dnSar1) through transduction by rAAV 24 h prior to parvovirus infection. Sec24 was inhibited by transfection of neutralizing antibodies (αSec24) 24 h prior to infection, αPKB served as control IgG. To measure potential signals from incoming capsids cells untreated cells were harvested at 4 h p.i. (B) Cellular extracts were fractionated by differential (density) centrifugation to separate organelles. Nuc, purified nuclei; HMF, large organelles; Cyt, cytosol. The LMF-fraction was fractionated by centrifugation through iodixanol gradients allowing cellular vesicles to be identified according to their density (fractions 8–11). The presence of progeny particles was determined by Southern blotting (revealing their single-stranded DNA). To determine the nature of PV virion-containing vesicles, cell lysates were treated with Triton X-100 (Triton) or proteinase K (Prot K) prior to fractionation. To rule out contamination from infecting particles, a fractionation experiment was performed after cell transfection with MVM infectious clone in the presence of neutralizing antibodies (Transf+B7 panel). The migration of Lamp2 (lysosomes/late endosomes) was determined by western blotting. (C) Cells were fixed with paraformaldehyde and stained for MVM capsids (green) and counterstained with lamin B (red). Infected cells of at least three individual experiments (>200 cells) were evaluated for the intracellular distribution of newly synthesized capsids. Grey columns: percentages of cells showing both a (peri)nuclear and cytoplasmic staining; hatched columns: percentages of cell showing a purely (peri)nuclear distribution. (D) Viral particles released into the medium. Medium and cell-associated virions were collected separately and quantified by dot-blot hybridization analyses for the DNA content and by standard plaque assays for infectious particles. Grey columns: MVM virion DNA; hatched columns H1-PV virion DNA; white columns: MVM plaque forming units.

Previous investigations have shown that progeny parvoviral particles become associated to vesicles in a gelsolin-dependent manner and are actively transported from the nuclear periphery to the plasma membrane [20]. To further characterize this pathway, we analyzed potential association of progeny particles in MVM infected A9 cells with cellular factors known to be involved in generation/loading of perinuclear vesicles. An obvious candidate for viral cargo shuttling out of the cell is the secretion pathway, which starts in the endoplasmic reticulum (ER), with sar1, sec23/24 and sec13/31-dependent formation of COPII vesicles. We therefore investigated the role of these factors in the association of progeny PV virions with vesicles and the further transport to the cell periphery. In a first step, we determined whether out-going MVM virions colocalize with sec13, sec23 and β-COP in infected A9 cells using laser scanning microscopy. Previous reports pointed that progeny particles are associated with lamp2, a protein present in the membrane of lysosomes and late endosomes/multivesicular bodies [20], [41]. We therefore used colocalizations with Lamp2 and mitochondria as positive and negative controls, respectively. The absence of any significant capsid fluorescence at 4 h p.i. was taken as a reference for the selective detection of newly produced virions after onset of virus propagation and not in-coming virus during the infection process. Furthermore, cell reinfection with progeny particles was prevented by addition of neutralizing antibodies to the medium after the initial infection. As illustrated in Fig. 1A, progeny PV particles showed clear colocalization with the cellular vesicle forming complex, giving a first hint that MVM virions may associate with cellular vesicles assembled at the ER membrane.

To functionally challenge this finding, we knocked down vesicle formation through transfection of neutralizing sec24 antibodies and expression of dominant-negative Sar1. Association of progeny particles with vesicles was determined by cell fractionation and monitoring single-stranded virion DNA presence in the corresponding biochemical fractions [20]. To confirm association of virus with membranes structures and to assess internalization into vesicles, cell extracts were treated, or not, with or triton X-100 (disruption of membranes, release of membrane associated proteins into cytoplasm) and proteinase K (elimination of all proteins that are unprotected by membrane structures) prior to fractionation. A 4 h p.i. time-point was chosen to discriminate egress from the infection process. Transfection experiments in the presence of neutralizing antibodies were performed to discriminate egress from (re-)infections. As shown in Fig. 1B and in agreement with the above hypothesis, viruses were found in the subcellular vesicle fractions, from which they were removed by exposing cell lysates to Triton prior to fractionation. This vesicle association was not detected when target cells were either transfected with sec24 neutralizing antibodies or if the cells expressed dominant-negative sar1 mutants. This suggests that MVM progeny virions become engulfed into vesicles by components of the cellular secretory pathway in the perinuclear ER. Similar results were obtained with the related rodent parvovirus strain hgH1-PV after infection of the human glioblastoma cell line NCH149, suggesting that vesicular egress of progeny virions is a general feature of rodent PVs.

We next studied the effect of functional sec23/24 and sar1 on PV egress. To this end, MVM-infected A9 cultures were analyzed for the proportion of cells supporting cytoplasmic transport of progeny virions, as determined by immunofluorescence microscopy and the amount of infectious particles shed into the medium supernatant. Cells lacking functional sec24 or sar1 were unable to transport progeny virions away from the nucleus, resulting in a marked increase in the proportion of cells showing only nuclear virus (Fig. 1C). While total virus production was not much affected by sec24 and sar1 knockdowns, the observed retention of virions in the (peri)nuclear area correlated with a significant reduction of virus release in the culture medium as determined by measuring the amounts of virion DNA (dot blots) and/or infectious virions (plaque assays) in the supernatant (Fig. 1D).

### Progeny virions are transported through directional movement of vesicles carriers from ER to the Golgi apparatus

**Figure 2:**
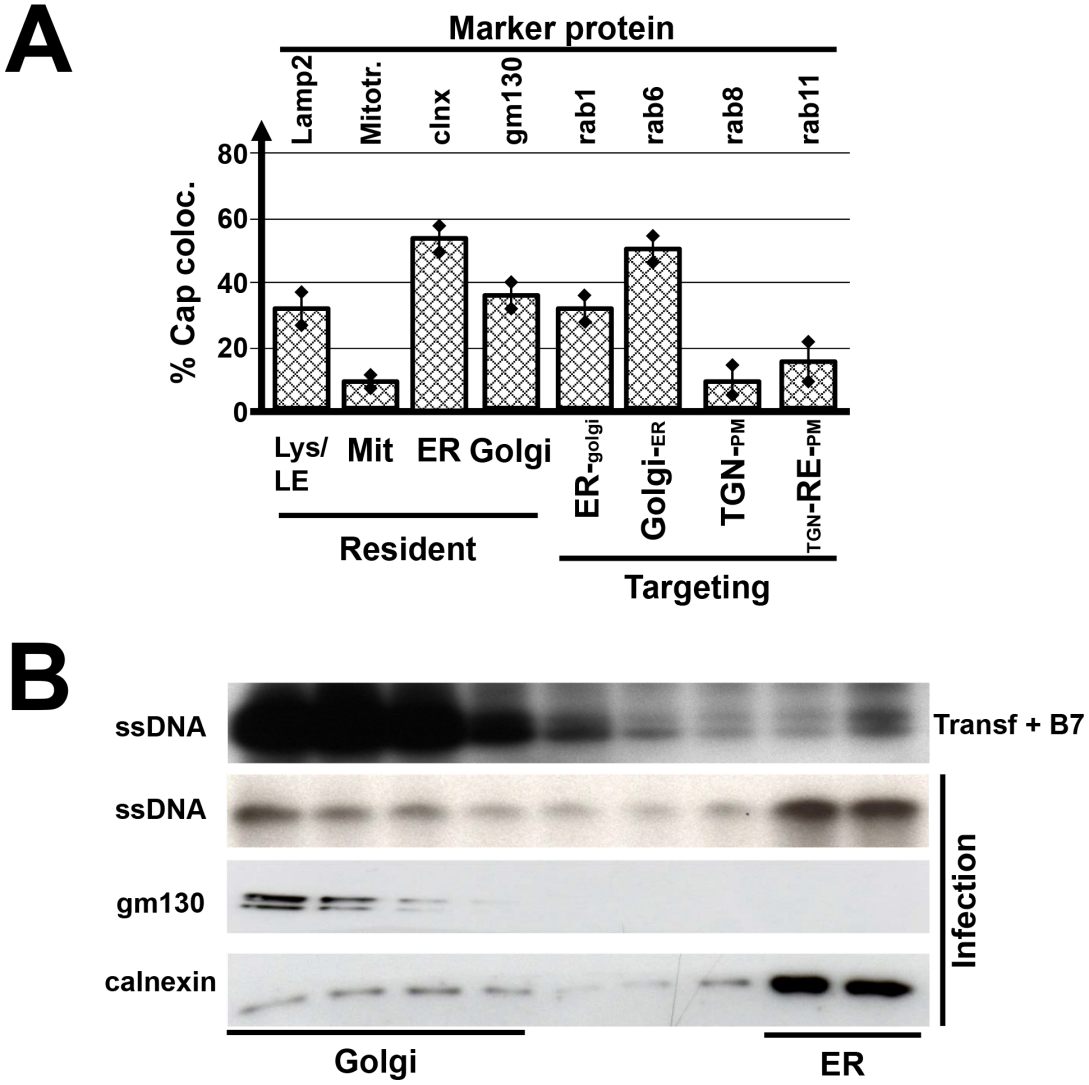
Colocalization of MVM virions with cytoplasmic marker proteins. A9 cells were infected with MVMp (30 pfu/cells) and analyzed 24 h p.i. for the presence of newly synthesized progeny virions. (A) Cells grown on spot slides were fixed with paraformaldehyde and analyzed by confocal laser scanning microscopy after double-staining using specific antisera for the indicated cell proteins and MVM capsids (Fig. S1). Virus neutralizing antibodies were added after the initial infection to prevent cell re-infections with progeny viruses. Colocalization was quantified by Image J analyzing infected cells from three individual experiments with >10 cells. Lys/LE, lysosomes/late endosomes (lamp2); Mit, mitochondria (mitotracker); ER, endoplasmic reticulum (calnexin [clnx]); TGN, trans golgi network; RE, recycling endosomes; PM, plasma membrane. (B) Cellular extracts were fractionated to separate golgi- versus ER-membrane structures by nycodenz gradient centrifugation. Progeny virions were detected by southern blotting measuring single-stranded virion DNA, the presence of gm130 (golgi) and calnexin (ER), respectively, by western blotting. To preclude interference from input viruses, a control was made by transfecting cells with MVM infectious DNA clone. Transfected cells were further incubated in the presence of neutralizing B7 antibodies to ensure that infection is limited to a single round and cell do not get reinfected with progeny viruses (Transf+B7 panel).

We next characterized the pathway followed by vesicles carrying PV progeny particles. Many enveloped viruses are transported through one or the other compartment of the cellular secretory pathway before mature virions are released by budding at the plasma membrane [1]–[6]. To determine the mode of parvoviral egress, we first examined the presence of MVM particles within cell organelles, and the association/colocalization of the particles with proteins involved in the targeting of intracellular vesicles. The latter proteins include the small GTPases, Rab that are located on the outside of vesicular membranes and are guiding the vesicles between subcellular compartments. Colocalisation with mitochondria (Mitotracker), ER (Calnexin, Sec23) and Golgi (gm130) as well as the small GTPases Rab1 (ER to Golgi), Rab 8 (Trans Golgi Network [TGN] to PM), and Rab11 (recycling endosomes) were investigated by confocal laser scanning microscopy. Rab6 which is involved in retrograde transport from Golgi to ER and is mainly detectable in the Golgi complex was used as a second marker for this organelle. It was shown previously that PVs become associated with Lamp2-containing vesicles (lysosomes, late endosomes/multivesicular bodies (MVB) [41]. Therefore, this marker served as a positive standard. (Re-)infections were inhibited by addition of neutralizing antibodies after the initial infections. As shown in Fig. S1 and summarized in Fig. 2A, strong colocalizations were observed between MVM particles and the ER resident protein calnexin, the Golgi complex marker gm130, and the small GTPases Rab1 and Rab6, known to control vesicular transfer between these compartments. Interestingly, significant co-staining was seen with Rab11 (TGN-RE-PM), while only background levels were obtained with mitotracker and Rab8 (TGN to PM). The presence of MVM progeny particles in ER and Golgi was then confirmed by biochemical fractionation of cellular extracts, separating ER membranes from the Golgi complex by ultracentrifugation on a nycodenz step gradient (Fig. 2B). Altogether these results strongly argue for the active transport of progeny PV particles through ER and Golgi.

**Figure 3:**
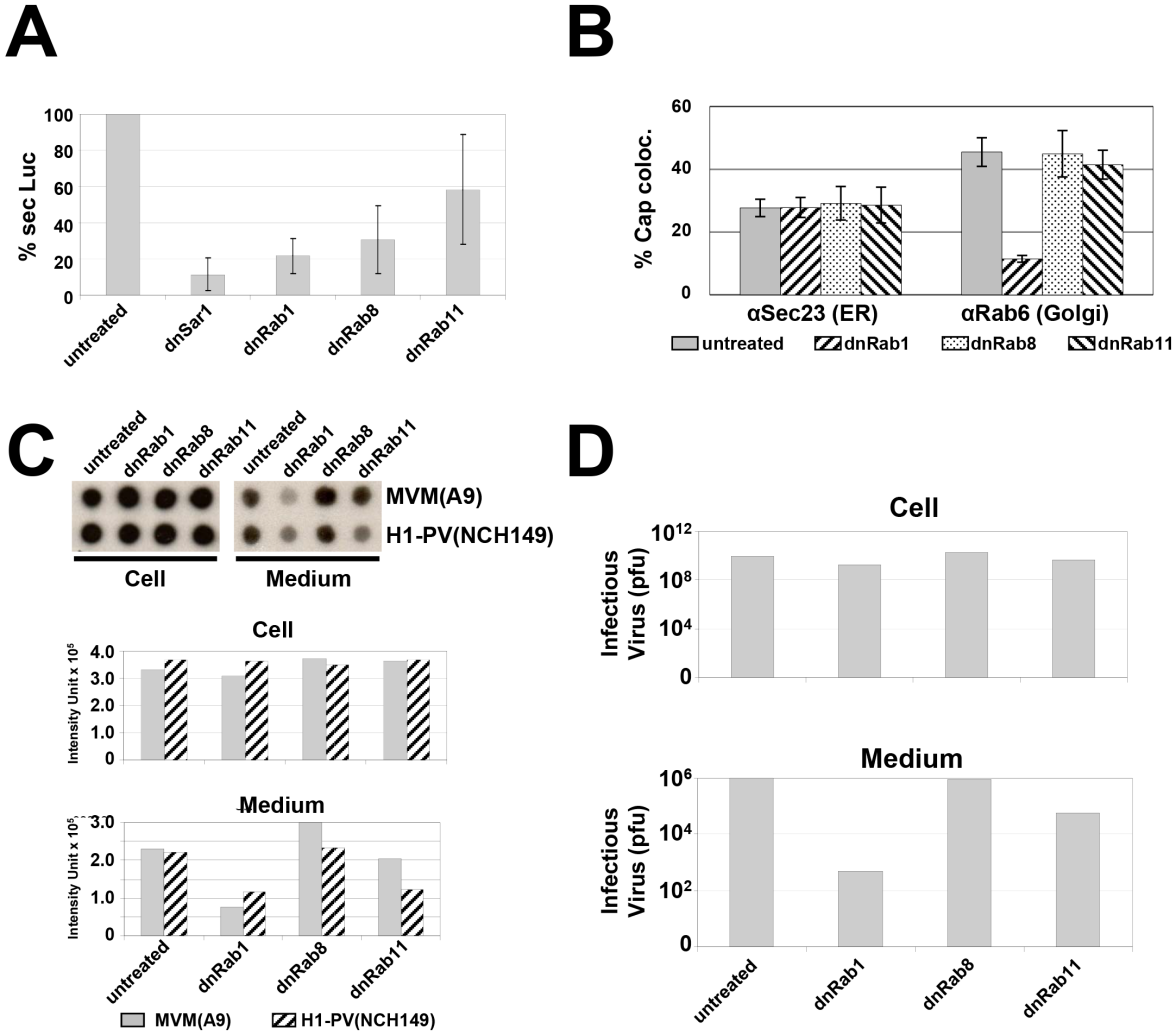
Cytoplasmic transport of progeny PV virions. (A) GLuc secretion through ER and golgi. A9 cells were transfected with pCMV-GLUC and transduced with rAAV:P38-dnRab1 (expressing dominant-negative rab1 (dnRab1)), rAAV:P38-dnRab8 (dnRab8) or rAAV:P38-dnRab11 (dnRab11), respectively, together with rAAV:P4-Transactivator. The amount of secreted GLuc was measured in the medium 72 h post transduction. Inhibition of GLuc secretion was determined in four independent experiments and mean values are displayed. rAAV:P38-dnSar1 served as a positive control. (B–D) A9 cells were infected with MVMp (30 pfu/cell), NCH149 cells with hgH1-PV (30 pfu/cells). When indicated, rab-protein functioning was inhibited by over-expression of the dominant-negative rab-variant (dnRab1, dnRab8, dnRab11), transduced by rAAV 24 h prior to parvovirus infection. Treated cells were processed at 24 h p.i. (B) Virus-neutralizing antibodies were added from 4 h p.i. on. Cells were fixed with paraformaldehyde and stained for MVM capsids together with either Sec23 (ER) or Rab6 (golgi), respectively, to monitor transport through these compartments ( Fig. S3). Colocalization was quantified by Image J analyzing 10 infected cells from three individual experiments. Grey columns: A9 untreated cells (untreated); hatched columns: A9 expressing dnRab1; dotted columns: A9 cells expressing dnRab8; back-hatched columns: A9 cells expressing dnRab11. (C, D) Viral particles released into the medium. Medium and cell-associated virions were collected separately and quantified (C) by dot-blot hybridization analyses for the DNA content and (D) by standard plaque assays for infectious particles. Grey columns: MVM virion DNA; hatched columns H1-PV virion DNA.

To functionally support these findings, secretion of intracellular proteins was inhibited by selective inactivation of distinct Rab proteins through (over-)expression of the corresponding dominant-negative mutants lacking GTPase activity [42], [43], [44]. To this end, A9 cells were transduced with rAAV virions expressing dominant-negative (and for controls functionally active) Rab proteins prior to MVM infection. To ensure efficient expression, the dnRab mutant genes were placed under the control of the parvoviral P38 promoter, and cells were co-transduced with rAAV:P38-dnRab and, in absence of the PV NS1 protein (i.e. in absence of PV infection), with a second recombinant AAV virus expressing a transactivator protein specific for this promoter (rAAV:P4-Transactivator). This synthetic transactivator protein is comprised of two distinct NS1 domains, the N-terminal site-specific DNA-binding domain (aa 1–275) and the C-terminal transactivator domain (aa 545–672) linked by GFP. Dimerization of this polypeptide takes place through an N-terminal GST-tag. In contrast to the natural PV NS1 protein, this polypeptide expressed from the PV P4-promoter proved to be non-toxic as assessed by measuring metabolic activity and cell lysis (Fig. S2A). Under these conditions, selective inhibition of the secretion pathway was first tested independently of MVM infection, using cells transfected with a plasmid expressing secreted Gaussia Luciferase (GLuc). Indeed, this enzyme is known to be transported by vesicles from ER to Golgi and further to the plasma membrane following the regular Rab1 and Rab8 dependent secretion pathway [45]. As shown in Fig. 3A, secretion of GLuc was efficiently blocked as a result of the expression of dnSar1 (driver for the formation of transport vesicles at ER exit sites), dnRab1 and dnRab8 but only marginal dnRab11, demonstrating the functional impact and specificity of these variant proteins. We next tested the effects of Rab1, Rab8 and Rab11 inhibition on the vesicular egress of MVM through ER (sec23) and Golgi (Rab6) by colocalization with corresponding marker proteins (Fig. 3B; Fig. S3), and on the release of progeny particles in the medium by DNA dot blot analyses (Fig. 3C) and plaque assays (Fig. 3D). As expected, none of the dominant-negative mutants prevented MVM particles from entering the ER compartment. In contrast, MVM transport from ER to Golgi, and virus secretion into the supernatant were strongly impaired upon expression of dnRab1. Moreover, a very limited reduction of secreted particles was observed with dnRab11, and nothing at all upon inhibition of Rab8, suggesting that PV particles take a different route from the Golgi apparatus to the plasma membrane as compared to GLuc.

### ERM family proteins are involved in the vesicular transport of progeny particles

**Figure 4:**
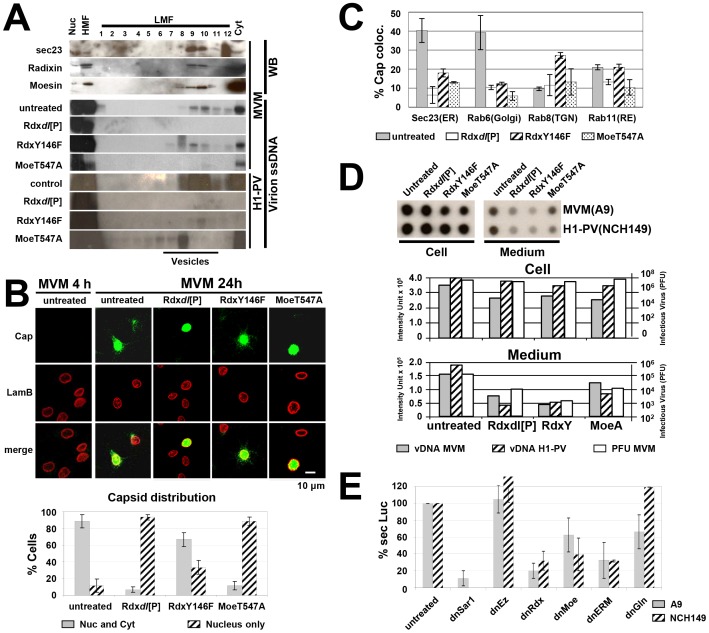
ERM family proteins contribute to the vesicular transport of PV progeny virions. A9 cells were infected with MVMp (30 pfu/cell), NCH149 cells with hgH1-PV (30 pfu/cells). When indicated, ERM (ezrin, radixin, moesin)-protein functioning was altered by over-expression of specific mutant protein variants (EzT566A (dnEz), RdxT564A (dnRdx), Rdx*dl*[P], RdxY146F (RdxY), MoeT547A (dnMoe)), transduced by rAAV 24 h prior to parvovirus infection. Treated cells were processed at 24 h p.i. (A) Cellular extracts were fractionated by differential (density) centrifugation to separate organelles. Nuc, purified nuclei; HMF, large organelles; Cyt, cytosol. Cellular vesicles were further purified from LMF by centrifugation through an iodixanol gradient. The presence of progeny particles was determined by Southern blotting (revealing their single-stranded DNA), the presence of cellular proteins by western blotting. Vesicular fractions are indicated. (B) Cells were fixed with paraformaldehyde at 4 (MVM 4 h) and 24 (MVM 24 h) h p.i., stained for MVM capsids (green) and counterstained with lamin B (red). Infected cells of at least three individual experiments (>200 cells) were evaluated for the intracellular distribution of newly synthesized capsids. Grey columns: percentages of cells showing both a (peri)nuclear and cytoplasmic staining; hatched columns: percentages of cell showing a purely (peri)nuclear distribution. Scale bar 10 µm. (C) Virus neutralizing antibodies were added after the initial infection to prevent cell re-infections with progeny viruses. Cells were fixed with paraformaldehyde and stained for MVM capsids together with either Sec23 (ER), Rab6 (golgi), Rab8 (TGN), or Rab11 (RE), respectively, to monitor transport through these compartments (Fig. S4). Colocalization was quantified by Image J analyzing 10 infected cells from three individual experiments. Grey columns: A9 untreated cells (untreated); white columns: A9 cell expressing Rdx*dl*[P]; hatched columns: A9 expressing RdxY146F; dotted columns: A9 cells expressing MoeT547A. (D) Viral particles released into the medium. Medium and cell-associated virions were collected separately and quantified by dot-blot hybridization analyses for the DNA content and by standard plaque assays for infectious particles. Grey columns: MVM virion DNA; hatched columns H1-PV virion DNA; white columns: MVM plaque forming units. (E) Involvement of ERM family proteins in GLuc secretion. A9 cells were transfected with pCMV-GLuc and transduced with rAAVs expressing dominant-negative mutants for ezrin (EzT566A), Rdx (RdxT564A), moesin (MoeT547A) or all three variants together (dnERM). The amount of secreted GLuc was measured in the medium 72 h post transduction. Inhibition of GLuc secretion was determined in four independent experiments and mean values are displayed. rAAV:P38-dnSar1 and rAAV:P38-GlnD565N transduced together with rAAV:P4-Transactivator served as controls.

Due to their impact on MVM plaque morphology in A9 cells, the ERM family proteins moesin (Moe) and radixin (Rdx) have been implicated in the spreading capacity of this parvovirus [38]. This observation, together with evidence of moesin being involved in endocytosis [46], led us to determine whether ERM proteins may play a role in the vesicular egress of progeny PV virions. In agreement with this possibility, we first showed that Moe and Rdx protein can be detected together with sec23 and virion DNA in vesicular fractions of MVM-infected A9 cells (Fig. 4A, 4 top panels). To put Moe and Rdx contribution to the test, we (over)expressed mutants thereof previously shown to interfere with endogenous protein functioning [38]. Impact on viral egress was then evaluated measuring virion DNA presence in vesicles (Fig. 4A, bottom), viral capsid intracellular distribution (Fig. 4B) and colocalization with cellular compartment marker proteins (Figs. 4C and S4), as well as progeny virion release into the medium (Fig. 4D). The dominant-negative moesin (MoeT547A) and radixin (Rdx*dl*[P]) mutants strongly interfered with the loading of progeny virions into cytoplasmic vesicles (Fig. 4A and C), resulting in a dramatic inhibition of virion release into the medium (Fig. 4D). Accordingly, viral capsids were hardly detectable in the cytoplasm under these conditions (Fig. 4B). In the presence of the functionally active RdxY146F mutant (consensus phosphorylation site for receptor tyrosine kinases [47]), MVM virions became associated with the vesicular fractions (Fig. 4A) and were readily found in the cytoplasm of infected cells (Fig. 4B). However, when vesicular egress through ER and Golgi was monitored, a strong decrease was observed in the colocalisation with the Golgi marker (Rab6, see Fig. S4), indicating the use of an alternative route seemingly involving secretory vesicles (increased Rab8 colocalization) and recycling endosomes (preserved Rab11 colocalization). Interestingly, in the presence of RdxY146F, progeny virions were not released efficiently into the medium (Fig. 4D), suggesting that virion transport through ER and Golgi plays an important role in the efficient release and spreading of PVs.

ERM proteins are known mediators of the interplay between actin cytoskeleton and membrane structures. The involvement of Rdx and Moe in the formation/loading of PV-containing vesicles raised the question, whether these proteins are selectively recruited by PVs or are genuine components of the cellular secretory machinery. This was investigated by expressing the secreted protein GLuc in mouse A9 and human NCH149 cells, and by determining the impact of over-expressing dominant-negative ERM variants on the proportion of enzyme secreted in culture supernatants in the absence of PV infections. As shown in Fig. 4E, inhibition of endogenous Rdx by RdxT564A strongly reduced the amount of GLuc in the medium from both cell cultures. A lesser but significant inhibition of GLuc secretion was also caused by dnMoe (MoeT547A). In contrast dnEz (EzT566A) failed to impair secretion. These data strongly argue for the essential role played by the ERM family proteins Rdx and Moe in the secretion of cellular proteins. The fact that dn mutant forms of these proteins inhibited in similar way PV egress gave further support to the involvement of vesicular transport in this process.

### Targeted vesicular transport of progeny virions is important for PV-induced cell lysis

**Figure 5:**
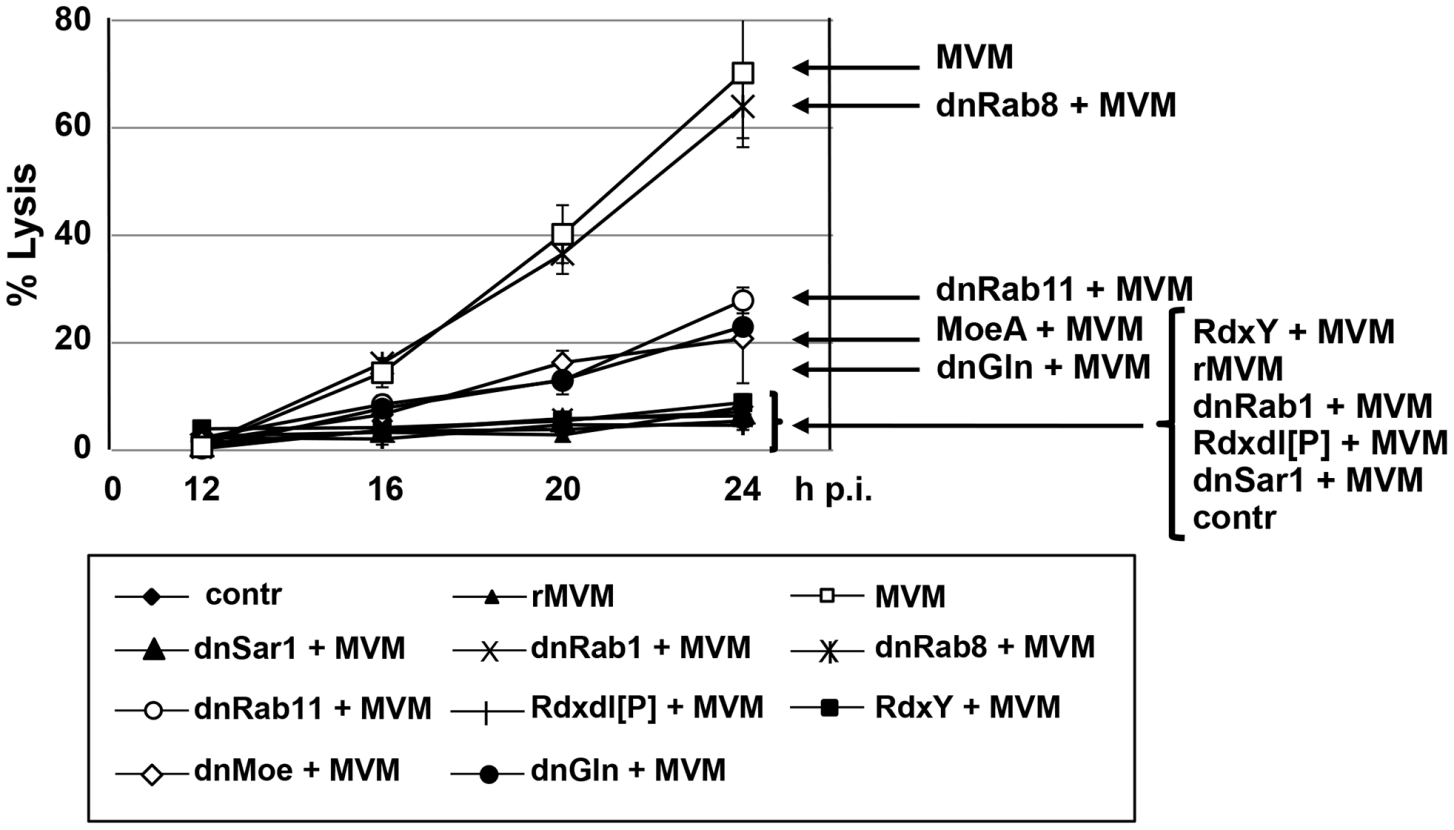
Vesicular transport mediates PV-induced cytolysis. A9 cells were infected or not with MVMp (30 pfu/cell). Subsequent reinfections were prevented by neutralizing progeny particles released into the medium by addition of B7 antibodies. When indicated, protein functioning was altered by over-expression of specific mutant protein variants (Sar1K38M (dnSar1), GlnY438A (dnGln), Rab1N25S (dnRab1), Rab8T22N (dnRab8), Rab11N25S (dnRab11), Rdx*dl*[P], RdxY146F, MoeT547A (MoeA), respectively) transduced by rAAV 24 h prior to parvovirus infection. rMVM denotes transduction of A9 cells with a viral vector deficient for progeny virion particles production due to substitution of the transgene GFP for the structural gene region. Contr stands for non-infected cells. Treated cells were incubated with propidium iodide for 30 min at indicated times p.i. and detected in transmitted light and fluorescent microscopy. PI positive cells and total number of cells from 3 fields of at least three individual experiments (>200 cells) were counted using Image J and proportion of lysed cells calculated.

A productive PV infection typically culminates in the rupture of the cell plasma membrane, as apparent from the generation of lysis plaques [15] and the release of intracellular polypeptides into medium supernatants [17]. This final lytic event clearly contributes to virus release and spreading, questioning the role of the vesicular egress of PV particles taking place prior to cell death. The above-mentioned impairment of progeny particle release upon down-modulation of vesicular transport (Fig. 1D, 3D, and 4D) made us wonder whether the latter transport may not only contribute to pre-lytic virion release but also control the lytic process which follows. To this end, time-course experiments were performed with MVM infected A9 cells under conditions where viral egress and release were modulated as shown in previous experiments. Permeabilization of the plasma membrane was then measured by propidium-iodide incorporation using fluorescent microscopy and image J software quantification. Mock-treated A9 cells served as negative controls. In addition, the relation of the lytic activity observed to the presence/absence of PV egress was tested by performing experiments with a recombinant MVM vector (rMVM). This vector produces similar amounts of NS1 as the wild type virus in a single-round infection, yet it fails to produce capsid proteins, and as a result, does not lead to virus egress [48]. Therefore, infection of A9 cells with rMVM was used to measure the mere lytic activity induced upon NS1 expression. The results are summarized in Fig. 5. MVM-induced cytolysis was strongly reduced when (i) the virus lacked the capacity for progeny particle production and in consequence transport to the PM (rMVM), or (ii) vesicular transport was either inhibited (dnSar1, Rdx*dl*[P], dnRab1) or by-passing the Golgi apparatus (RdxY146F). Inhibition of particle loading (dnGln, MoeT547A) resulted in an intermediate phenotype as apparent from the delayed and significantly reduced PI incorporation. This was also the case of cells expressing dnRab11, correlating with a reduction of virus release into the medium (see Fig. 3C and D), presumably due to inhibition of the Golgi-TGN-RE pathway. In contrast, no or little effects on cell permeabilization were seen with dnRab8, which did not impair egress of PVs (Fig. 3C and D). Altogether these results suggest that vesicular transport of progeny particles through the Golgi complex plays an important role in the induction of cytolysis and to ensure the further release of progeny particles at the end of infection.

### Progeny MVM particles become modified during egress through ER and Golgi, resulting in a gain of infectivity

**Figure 6:**
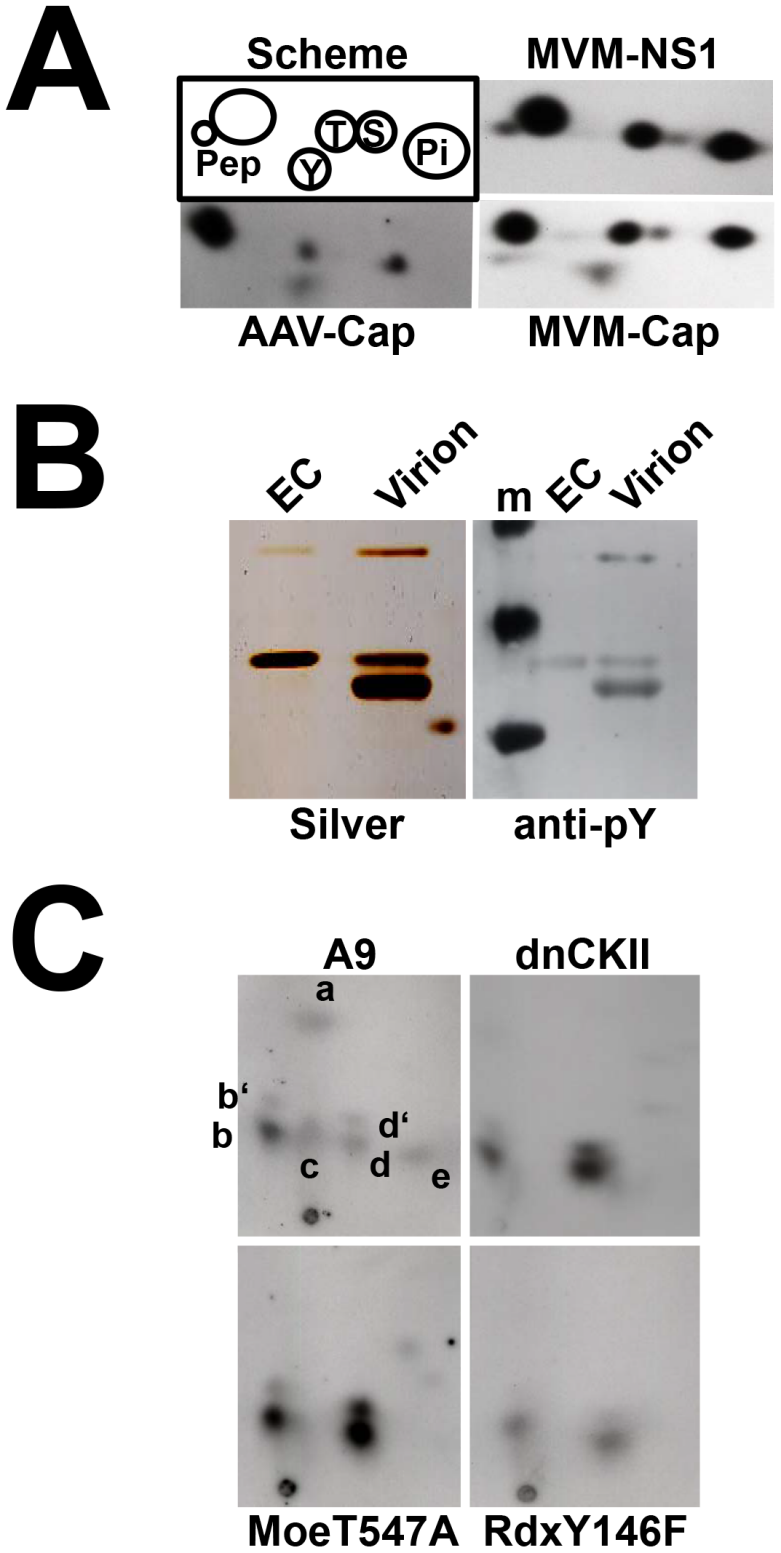
Modification of PV-virions during vesicular transport through ER and golgi. (A) Phospho amino acid analysis of MVM NS1, progeny virions and AAV capsids. A9 cells were infected with MVMp (30 pfu/cell), HeLa cells with wild type AAV2 (1000 IU/cell) in the presence Ad5, and subjected to metabolic ^32^P-labeling with orthophosphate at 24 h p.i. Newly synthesized MVM virions, MVM NS1 and AAV capsids were isolated by immunoprecipitation, purified on SDS-PAGE and analyzed for their phosphor amino acid composition by 2D-electrophoresis. A scheme (upper left corner) denotes the migration of the different phosphorylated products. (B) Double-purified MVM virions and empty capsids (EC) were characterized by SDS-PAGE followed by silver-staining (left panel) and western blotting to detect phosphor-tyrosine residues (right panel), respectively. (C) A9 cells or derivative cell lines thereof expressing dominant-negative CKIIα (CKII-E81A), dominant-negative moesin (MoeT547A), or mutant radixin Y146F (RdxY146F), were infected with MVM (30 pfu/cell), and ^32^P-labeled with orthophosphate at 24 h p.i. Newly synthesized capsids were isolated by immunoprecipitation, purified by SDS-PAGE and subjected to tryptic phosphopeptide analyses. Resolved phosphopeptides are labeled a through e.

Vesicular trafficking through the Golgi apparatus raises the intriguing possibility of assembled PV particles undergoing maturation and gaining full infectivity in this compartment. This prompted us to first determine whether the egress process was accompanied with post-assembly phosphorylations of MVM capsids. Unlike NS1 whose phosphorylation is targeted at serine and threonine residues [49], MVM (as AAV2) progeny particles purified from infected cells were also phosphorylated at tyrosines (Fig. 6A&B). After metabolic ^32^P-labeling, these phosphorylations resolved into a complex pattern of seven distinct tryptic phosphopeptides in two-dimensional electrophoresis/chromatography analysis (Fig. 6C). To determine the impact of vesicular transport on MVM capsid phosphorylation, effector protein variants known to inhibit viral egress (CKIIαE81A [20], MoeT547A, and RdxY146F [see above]) were tested for their effects on the capsid tryptic phosphopeptide profile. As shown in Fig. 6C, the inhibition of vesicular egress or its re-routing away from the Golgi compartment, led to remarkable changes in the capsid phosphorylation pattern, in particular the characteristic loss of three distinct phosphopeptides (a, c, and e). These data provide strong evidence of parvoviral capsid modifications during egress through ER and Golgi.

**Figure 7:**
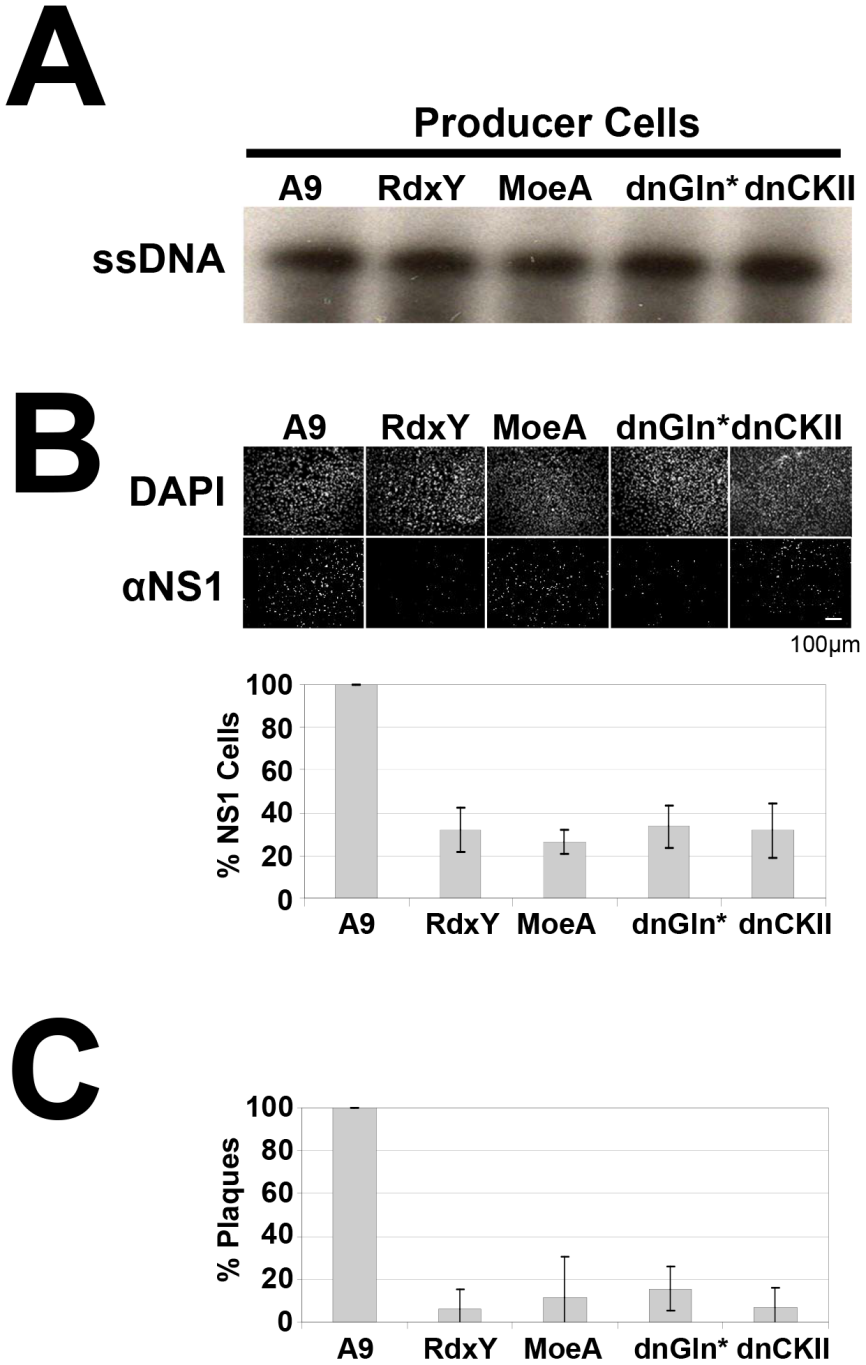
Maturation of progeny MVM virions during vesicular transport through ER and golgi. (A) A9 cells or derivatives thereof expressing mutant RdxY146F (RxY), dominant-negative MoeT547A (MoeA), dominant-negative gelsolin GlnD565N (dnGln*), or dominant-negative casein kinase II CKIIαE81A (dnCKII), were infected with MVMp (0.3 pfu/cell) to produce virus stocks. (A) CsCl-purified virions were matched and analyzed for their single-stranded DNA content by southern blot analyses. (B, C) Titration experiments with matched amounts of single-stranded virion DNA were performed in A9 cells and analyzed for (B) the proportion of NS1-positive cells by immunofluorescence staining (scale bar 100 µm) in three individual experiments measuring >200 cells and (C) the proportion of infectious particles by standard plaque assays in A9 cells in three individual experiments.

We next determined the impact of vesicular egress on viral infectivity. Virus stocks were produced both in parental A9 cells, and in stable A9-transfectants, which have been previously shown to suppress vesicular egress of progeny PV particles due to the expression of variant cellular proteins (GlnY438A, CKIIαE81A [20], RdxY146F, MoeT547A [see above]). Virus stocks were purified on CsCl-gradients to remove empty capsids, matched for their ssDNA content (Fig. 7A) and tested for their infectivity in A9 cells by counting both NS1-positive cells (Fig. 7B) and lysis plaques (Fig. 7C) in infected cultures. Progeny particles that had transited (at least in part) through the Golgi complex in parental A9 cells proved to be significantly more infectious (lower particle (DNA) to infectivity ratio) than their counterparts that had bypassed this compartment (RdxY146F, MoeT547A, GlnY438A, CKII-E81A). For the same amounts of viral genome, virus produced in the latter four cells gave rise to 3 to 4 times less NS1-positive cells and up to 10 fold fewer plaques than virus produced in vesicular egress-proficient cells (A9). Together with the above finding that egress through ER and Golgi is accompanied by capsid modifications, these results suggest that the transport pathway to the plasma membrane represents an important step in the maturation of progeny virions.

## Discussion

Progeny virion shuttling from the nucleus, the site of parvovirus replication and assembly, to the plasma membrane is an important step in virus release and cell to cell spread. The present analysis shows that the release of non-enveloped PVs is not a mere consequence of the cellular lytic burst occurring at the very end of the viral life-cycle but involves a pre-lytic active vesicular transport of virions. This transport makes virions transit through ER and Golgi, and appears to have two major implications: the maturation of progeny particles into fully infectious virions, and the induction of cytolysis with the ensuing virus spread. For this transport process, PVs usurp cellular components allowing progeny virion engulfment into COPII vesicles at the ER and moving through the Golgi to the plasma membrane under control of small Rab GTPases. This vesicular trafficking is a general feature of rodent parvoviruses as it was demonstrated for both, the mouse virus MVM and rat virus H-1PV in mouse and human cancer cells, respectively.

Besides known cellular components of vesicular transport (Sar1, sec13/31, sec23/24), the ERM family proteins radixin and, to a minor extent, moesin were found to be involved in the formation of PV-loaded COPII-vesicles. Given their known mediator function between actin and membrane structures [40], we hypothesize that ERM proteins may control the engulfment of viral cargo into membrane structures. In keeping with this possibility, we recently reported that progeny PV particles associate with actin fragments, and that the actin-severing protein gelsolin controls PV loading [20]. Interestingly, the present results indicate that, radixin and (at least in human cells) moesin are essential players not only for PV egress but for the secretion of GLuc as well. This speaks for the fact that ERM family proteins are not simply involved in PV virion sensing, but play a general role in the COPII vesicle-mediated secretion. In contrast, the actin-processing protein gelsolin, whose knock-down had no impact on GLuc secretion, seems to be recruited specifically to promote (parvo)virus egress and is apparently not required for cellular secretion in general.

During their traffic from the nucleus to the PM, PVs appear to transit through the Golgi compartment, as evidenced by biochemical fractionations and co-staining data. This is unexpected, since in contrast with enveloped viruses, PVs do not acquire, during this transit, a lipid envelope harboring proteins mediating cell attachment and/or entry. However, passage through the ER-Golgi pathway still proved to be important for PV maturation in that capsids gained post-assembly modification such as phosphorylations. These capsid modifications did not occur under conditions inhibiting the vesicular secretory pathway. MVM viruses lacking the Golgi-associated capsid modifications were less efficient at infecting naïve cells as compared with viruses produced in cells with an intact secretory pathway. This is not without precedent for non-enveloped lytic viruses. Indeed, tyrosine phosphorylations at the capsid surface of the defective parvovirus AAV also seem to control the infectivity of this agent [50]. Furthermore, other viruses become processed after assembly. For instance, assembled polioviruses present in autophagosomes, were shown to mature upon acidification of these vesicles prior to their fusion with lysosomes. This acidification induces the cleavage of poliovirus capsid proteins, an essential step for these virions to gain full infectivity [51]. Interestingly, MVM and H-1PV virions were shown to colocalize with the late endosomal/lysosomal marker Lamp2 (see also below). This leads us to speculate that the maturation of these viruses may involve the induction of additional post-assembly capsid modifications in these compartments besides the above-mentioned Golgi-associated phosphorylations.

These observations raise the question how parvoviruses get sorted into the intracellular cisternae system. A number of enveloped viruses (e.g. Filo-, Retro- Rhabdo-, Arena-, Paramyxo viruses) transit through multi vesicular bodies (MVB) during their transport to the PM. A common feature of these viruses consists in the presence in their structural proteins, of (a) short recognition motif(s) facilitating their interaction with cellular factors that participate in MVB sorting. In the case of HIV-1, the PTAP-motif of Gag interacts with Tsg101, thereby inducing the recruitment of the ESCRT1 complex which constitutes the sorting machinery for incorporation of ubiquitylated proteins into MVBs. Thus, this interaction promotes entry of viral particles into MVBs during HIV-infection [52]–[55]. Other motifs known to mediate vesicular uptake include YPx(n)L and PY (PPxY), which bind to the cellular factors AIP1/Alix and Nedd4 ubiquitin ligase, respectively [56], [57]. It is noteworthy that PPxY motifs are present in the parvovirus VP1 protein. Together with the strong and weak colocalization of PV capsids with the late endosome markers Lamp2 [41], [58], and Rab11 (present study), respectively, this feature raises the possibility of late endosomes/MVBs playing a role in the transport of PVs from the Golgi to the PM.

Besides contributing to virus intracellular trafficking, vesicular transport of progeny PV particles through ER and Golgi apparently serves additional purposes. Importantly, this vesicular transit was found to result in post-assembly modification (in particular phosphorylation) of capsids, and in a marked increase in virion infectivity. Furthermore, vesicular egress proved to have an impact on the still poorly understood mechanism of PV-induced cytolysis. The present results indicate that progeny virus transport through ER and Golgi up-regulates this process. This is apparent from the delay in or inhibition of infected cells lysis upon alteration of the virus egress route. The mechanism underlying this dependence of cytolysis on vesicular PV transport remains a matter of speculation. It may be traced back to the co-transport of cellular or viral products involved in cytolysis, which become engulfed into virion-containing vesicles, are brought to the PM and cause its permeabilization. Besides cellular or viral phospholipases, the viral NS1 protein is an intriguing candidate for this process, since it appears to exert lytic activities upon (prolonged) ectopic expression [59] and is known to be present in cellular vesicles [20], [41]. At late stages of infection, the vesicular co-transport of cytolytic factors might not be required anymore since the cytoskeletal collapse [33], [36] would allow direct access of cellular and viral lytic factors to the PM. This would explain the delayed cell lysis and small plaque phenotype observed after PV infection of cells deficient in ER-Golgi vesicular transport.

As a whole, the present study revealed not only the subcellular pathway followed by progeny PVs to exit infected cells, but also the unexpected role played by this vesicular egress in the interconnected process of cytolysis, virus maturation and spread. The latter processes are of particular importance in the context of the current development of PVs as oncolytic agents for anti-cancer therapeutic applications [60]. It is noteworthy in this regard that tumor cell killing by parvoviruses is immunogenic [61], resulting in an immune bystander effect that takes over from the initial direct oncolytic effect of the viral treatment to complete eradication [62]. It will be of great interest to determine whether the hereby reported vesicular egress of parvovirus particles also contributes to the display and/or release of tumor-associated antigens and/or damage/pathogen-associated molecular patterns that stimulate the immune component of PV oncosuppression.

## Materials and Methods

### Antibodies and reagents

#### Primary antibodies

Sec13, Sec23, calnexin (Abcam ab22595), gm130, rab1A (Sta. Cruz Biotechnologies sc26541), rab6 (Sta. Cruz Biotechnologies sc310), rab8A (Sta. Cruz Biotechnologies sc26578), rab11A (Abcam ab65200), radixin (Sta. Cruz Biotechnologies sc6408), moesin (Sta. Cruz Biotechnologies sc6410) antibodies as well as antibodies against Flag- (M2) and myc-tag (Sigma: F7425, M5546), lamin B (Sta. Cruz Biotechnologies: M-20 sc6217), Lamp2 (Abcam ab37024), Sar1 (Abcam ab77473), Sec23 (Sta. Cruz Biotechnologies sc20789); Sec24 (Sta. Cruz Biotechnologies sc169279) and PKB (Sta. Cruz Biotechnologies sc1692791618). Rabbit antiserum recognizing the MVM NS1 (αNS1_C_) [20], antiserum recognizing VP2 (αVP2) [20], and monoclonal anti-capsid antibody B7 [63] were described previously. Phosphotyrosine specific mouse monoclonal antibodies (12CA5) were kindly provided by Prof. Dr. Angel Alonso (German Cancer Research Center).

#### Secondary IgGs

Horseradish-peroxidase-conjugated (HRP-conjugated) anti-rabbit and anti-mouse IgGs (Promega), HRP-conjugated anti-goat IgGs (Sta. Cruz), Alexa fluor fluorescent-dye-labeled IgG (Dianova and Invitrogen). *Others:* Protein G sepharose beads (Pharmacia Amersham), [^32^P]-labeled α-dCTP (Perkin Elmer), [^32^P]-orthophosphate (MP Biomedicals).

### Previously described and functionally characterized effector constructs

#### Protein kinases

Flag-tagged CKIIαE81A (dominant-negative) [36], [37].

#### ERM-family proteins

FL-EzT566A (dominant-negative), FL-RdxT564A (dominant-negative), FL-RdxT564E (constitutive-active), FL-Rdx*dl*[P] (dominant-negative), FL-RdxY146F (active), FL-MoeT547A (dominant-negative) [38].

#### Gelsolin

FL-Gln Y438A and FL-D565N [20].

### Site-directed mutagenesis and cloning procedures

Site-directed mutagenesis was performed by single or chimeric PCR, cloned into pCR2.1 vectors (Invitrogen) and verified by sequencing [64].

#### Effector genes

dnSar1 was obtained by the substitution of lysine at position 38 by a methionine using following primers: Sar1K38M-F: ggataatgccggga**tg**acaactttgctacac and Sar1K38M-R: gtgtagcaaagttgt**ca**tcccggcattatcc. The dnRab1 mutant corresponds to the substitution of serine 25 by an asparagine [42] using following primers: Rab1S25N- F: gttggaaag**aac**tgccttctcc and Rab1S25N-R: ggagaaggca**gtt**ctttccaac. The dnRab8 mutant corresponds to the substitution of serine 29 by an asparagine [43] using following primers: Rab8T22N- F: ggggtggggaaga**a**ctgtgtgctg and Rab8T22N-R: cagcacacag**t**tctcccccacccc. The dnRab11 mutant corresponds to the substitution of serine 25 by an asparagine [44] using following primers: Rab11S25N- F: ggtgttggaaag**aat**aatctcctg and Rab11S25N-R: gagatt**att**ctttccaacacc. All four variants were cloned in order to express an N-terminal myc tag using an N-terminal primer harboring the respective sequence in frame with the original start codon.

#### Fusion polypeptide serving as a non-toxic transactivator for the MVM P38-promoter

Domain fusions were performed with 40mer primers consisting of 20 nts overlapping sequences of each domain. Assembly of the different domains was achieved by chimeric PCR as previously described for the adaptor constructs mimicking MVM NS1oncotoxicity [37]. In brief: First rounds of PCR were performed to construct produce the individual domains with overlapping sequences to the neighboring domains: GST: (a) *gtttaaac*atgtcccctatactaggttattggaaaattaa with (b) gagtaagcatttccagccatcagatccgattttggaggat; NS1-DNA_B_ (aa 1–275): (c) atcctccaaaatcggatctgatggctggaaatgcttactc with (d) tcctcgcccttgctcaccattctgccgcgcttagtttcct; GFP: (e) aggaaactaagcgcggcagaatggtgagcaagggcgagga with (f) ttagcacagtagcttgccatcttgtacagctcgtccatgc; NS1-TA (aa 545–672): (g) gcatggacgagctgtacaagatggcaagctactgtgcaaa with (h) *gcggccgc*ttagtccaagttcagcggctcgctgaagtctt. The second round PCR combined the GST with NS1-DNA_B_ (aa 1–275) using primer (a) and (d), in a separate PCR, GFP with NS1-TA (aa 545–672) using primers (e) and (h). The third round combined the two fusion-constructs from the second PCR with each other to produce GST∼NS1-DNA_B_∼GFP∼NS1-TA with the primer pair (a) and (h) (Fig. S2A).

#### Production of expression constructs for generating stably transfected cell lines

MVM NS1-inducible expression vectors were constructed from plasmid pAAV2:pP38-GFP, where Myc/Flag-tagged protein variants were transferred from pCR2.1 vectors, replacing the GFP reporter gene [64]. *rAAV2:P4-X and rAAV2:(pA)P38-X constructs*. pAAV:P4-GFP contains the GPF-gene under the control of the MVM flanked by multiple cloning sites. This allows easy replacement with candidate gene-sequences [NcoI,PmeI,XbaI,Eco47III]-GFP-[EcoRV,HindIII,XhoI,StuI,NotI]. pAAV2:(pA)P38-GFP contains the same GFP cassette under the control of the NS1-inducible (H1-PV) P38 promoter. Potential promoter activity through the left-end ITR was blocked by insertion of the MVM poly(A) sequence at H1-NS1 position nt1493. It was constructed as follows: pTRH1-Gfp [65]was first cleaved with PmeI/Bst1107I, ligated. The PmeI/Not cleaved GFP cassette was then inserted into the StuI/NotI cleaved P4-less pTRH1-Gfp. Finally the PCR-generated MVM poly(A) sequence was inserted into the NheI-cleaved construct and the correct orientation was checked by a BamHI-digest due to the presence of a new BamHI-site at the left-end of the poly(A) sequence. Effector genes, cut blunt-end (N-terminus) and NotI (C-terminus) were then inserted into the Eco47III- and NotI-cleaved pAAV2:P4-X or pAAV2:P38-X vectors, respectively, to generate the corresponding pAAV-P4/P38-X constructs.

#### Reporter construct pCMV-GLuc

The Gaussia Luciferase expressing construct under the control of the CMV promoter was generated by transferring the GLuc gene from the pGLuc-Basic vector (New England Biolabs) as a PCR-derived EcoRI-fragment into similarly cleaved pCR3.1 (Invitrogen).

### Cells and viruses

All cell lines were maintained as monolayers in DMEM containing 10% FCS. Stable transfectants were generated with pP38-X and the selection plasmid pSV2neo or pTK-hyg at the molar ratio of 25∶1. Colonies were pooled after growth under selection and frozen stocks prepared. Experiments were performed in the absence of drugs [64]. MVM was propagated in A9 cells. Human-glioma propagation-competent hgH1-PV strain was obtained after serial passaging in NCH149 cells and propagated in NB324K cells [20]. To produce virus stocks under conditions lacking vesicular egress through ER and Golgi, A9 derivative cell lines A9:P38-FL-GlnD565N [20], A9:P38-CKII-E81A [20], [36], A9:P38-MoeT547A, and A9:P38-RdxY146F [38], respectively were infected with 0,03 PFU/cell MVMp and harvested upon detection of severe CPE. All virus stocks were purified after freezing and thawing over CsCl density gradient [39] and quantified by their amount of single-stranded DNA by southern blotting and standard plaque assays. PV-infection was limited to a single round by addition of either 0.5 U/ml neuraminidase (Sigma) to remove sialic acid from the cell receptor 1/200 dilution of neutralizing antibody B7 as from 4 h after the initial infection (Supplement S5)o Recombinant adeno-associated viruses (rAAVs) were generated in 293T cells in absence of Ad5 by co-transfection with pAAV-P4-X and pDG, purified by iodixanol step gradients and the genomic titers determined by dot blot hybridization [66]. Transduction efficiencies were determined by immunofluorescence microscopy measuring the proportion of trans-gene expressing cells. Toxicity was assessed by measuring metabolic activity through mitotracker incorporation, and cell lysis through PI-staining (Supplement Fig. S6A, B).

### Antibody transfection

When indicated, transfection with Sec24-neutralizing antibodies and for a control PKB antibodies were performed 4 h prior to virus infection, with 10 µg IgG and 20 µl Ab-DeliverIN (OZ Biosciences) per 5×10^5^ cells. To prevent secondary rounds of infection, the cells were treated with 5 U/ml neuraminidase (Sigma) 4 h p.i. Transfection efficiency and impact on virus infection were examined by immunofluorescence microscopy measuring the proportion of antibody transfected cells and the amount of NS1-expressing cells, respectively (Supplement Fig. S6C).

### Determination of infectious titers by standard plaque assays [15], [17]

To determine the presence of infectious particles monolayer cultures (A9 for MVMp, NB324K for hgH1-PV) were seeded at a concentration of 10^5^ cells per 60 mm^2^ dish, infected 24 h later and covered with a Bacto-agar overlay. After incubation for 6 days, cultures were stained for 18 h by addition of neutral-red containing Bacto-agar. Stained cells were fixed on the plates with formaldehyde after removing the agar overlay.

### Detection and quantification of viral parvoviral DNA by southern blot or dot blot hybridization

Accumulation of parvoviral DNA species (monomer/dimer replication intermediates and single-stranded virion DNA) were determined by Southern blotting [49], total viral DNAs by dot blot hybridization. At the indicated times p.i., medium was removed and kept separately. Adherent cells were washed, harvested in DMEM without serum by scraping from the dish, and collected by centrifugation. Medium- and cell-associated virions were quantified, after repeated freezing and thawing, in standard plaque assays (see above) or after proteinase K digest by southern and/or dot blot hybridization: Cell associated viral DNAs were harvested in vTE, digested with proteinase K and total DNA was shared through syringe. To determine the amount of virion DNA in purified virus stocks serial dilutions were performed, medium associated viral DNAs were harvested and adjusted to “vTE-conditions” and digested by proteinase K. To detect different viral DNA species, samples were analyzed by agarose gel electrophoresis and transferred onto nitrocellulose membranes. Total viral DNA was transferred to nitrocellulose using a dot-blot apparatus. Viral DNA was detected by hybridization with a ^32^P-labeled probe corresponding to nts 385–1885 of the NS1-encoding region of MVM DNA and when indicated quantified after autoradiography with GelQuant.NET software.

### Western blot analyses [20]

Cellular extracts were produced in Co-Ip buffer and cleared by centrifugation. Protein extracts were then fractionated by discontinuous SDS-PAGE and blotted onto nitrocellulose membranes. Blocking was performed in 10% dry milk/PBS or for phosphospecific antibodies in 2% casein,10 mM Tris pH 8.0, 150 mM NaCl, 1 mM EDTA, 0.1% Triton X-100 for 18 h. Staining with horseradish-peroxidase-conjugated secondary antibodies for 1 h followed by chemiluminescence detection (Amersham).

### Quantification of Gaussia Luciferase (GLuc) in cells and medium supernatants

10^5^ cells were transfected with 5 µg pCMV-GLuc expressing Gaussia Luciferase under the control of the CMV promoter using 25 µl lipofectamin in OptiMEM. Medium was changed 4 h post transfection, cells were transduced with 10^4^ genomes/cell of the indicated rAAVs and further incubated for 72 h. Supernatants were collected and the cell monolayers were lyzed in 25 mM Tris pH 7.5, 250 mM NaCl, 5 mM EDTA, 0.1% NP-40. G-Luc activity t in medium and cell lysates was then determined upon addition of 5 µl GLuc substrate coelenterazine (Biotium, Inc) by fluorescence (465 nm) detection with a luminometer.

### Immunofluorescence microscopy [20]

Cells were grown on spot slides (Roth). Cultures were fixed with 3% paraformaldehyde and permeabilized with 0.2% Triton X-100. Specimens were pre-adsorbed with 20% FCS, incubated with primary antibodies, and stained with specific Alexa Fluor 488 or/and 564 conjugated anti-species antibodies. DAPI (10 µg/ml) was added to the secondary antibody solutions. Analyses were performed with a Leica DMIRBE confocal microscope (63× lens, laser: red 543 nm, green 488 nm) and Powerscan software or with an Olympus Fluoview FV1000 confocal microscope (63× lens, laser: green 488, red 594 nm, far red: 633 nm) presenting a single slice of a stack. Quantitative analyses and mean colocalizations were calculated with ImageJ software. For PI staining, cells were incubated with propidium iodide (1 µg/ml) for 30 min at 37°C. Pictures of living cells were taken on an inverted LeicaDFC350FX microscope using transmitted and fluorescent light, PI positive and the total number of cells counted using ImageJ and the proportion of PI positive cells calculated.

### Biochemical fractionation of cell extracts

#### Separation of nuclear, mitochondrial, and vesicular fractions from the soluble cytosol [41]

Nuclear components were obtained by pelleting at 800 g and further purification through 1 M sucrose. The 800-g supernatant was centrifuged at 2500 g to pellet large organelles like mitochondria in a “heavy mitochondrial fraction” (HMF), and the supernatant was centrifuged at 17000 g to pellet smaller organelles, including vesicles, in a “light mitochondrial fraction” (LMF). The final supernatant represented the soluble cytosolic fraction. The LMF-fraction was used to determine association of newly synthesized virions with vesicles. The LMF pellet suspended in hypotonic buffer was added to 50% iodixanol/142 mM sucrose (2∶1 v/v), and the components were separated according to their density by centrifugation for 4 h at 4°C in a self-forming gradient in a vertical rotor at 380,000 g. 12 fractions were collected from the top, volume-matched with the nuclear, HMF, and cytosolic fractions, and analyzed individually by Southern and western blotting.

#### Separation of endoplasmic reticulum (ER) and golgi membranes

Separation of ER and golgi proteins was essentially achieved as previously published [67]. 2×10^7^ A9 cells were washed, scraped into ice-cold PBS and centrifuged at 70×g for 10 min at 4°C. The cell pellet was suspended in 500 µl homogenization buffer (50 mM Tris ph7.5, 130 mM NaCl, 30 mM KCl, 3 mM EDTA) and passed multiple times consecutively through a syringe (5×G22 and 5×G24). Nuclei were then separated by centrifugation at 1000×g for 5 min and discarded. The remaining supernatant was adjusted to 2 ml and centrifuged through a nycodenz step-gradient (2 ml 30%, 1.33 ml 20%, 1.33 ml 15%, 1.33 ml 10%, 1.33 ml 7.5%, 1.33 ml 5%, 1.33 ml 2.5% (w/v nycodenz in homogenization buffer) at 33,000×g for 15 min using a SW41 rotor (Beckman instruments). Nine fractions of 1.33 ml were collected from the top and analyzed by southern and western blotting respectively.

### Metabolic labeling, purification, phosphopeptide analyses and phosphor amino acid analyses [49], [68]

A9 cell cultures were subjected to labeling medium for 4 h (0.1 nCi/cell of [^32^P]. Labeled cells were harvested and the respective proteins isolated by immunoprecipitations and purified by SDS-PAGE. ^32^P-labeled proteins blotted on PDF-membranes were revealed by autoradiography, excised and digested trypsin. Tryptic peptides were analyzed on thin-layer cellulose plates (Merck) in two dimensions, first by electrophoresis in a pH 1.9 buffer, and then by chromatography in phosphochromatography buffer. Phospho amino acid analyses were performed with excised ^32^P-labeled protein bands. Individual labeled amino acids were obtained by hydrolization in 6 M HCl at 110°C for 1 h and phosphoserine, phosphothreonine, and phosphotyrosines were separated by thin-layer electrophoreses in two-dimension using ph1.9 and ph3.5 buffers, respectively.

## Supporting Information

Figure S1 **A9 cells grown on spot slides were infected (MVM 24 h) or not (A9) with MVMp (30 pfu/cell) and further incubated under conditions neutralizing progeny particles released in the medium.** Cells were then fixed with paraformaldehyde 24 h p.i., and analyzed by confocal laser scanning microscopy after double-staining using specific antisera for the indicated cell proteins (red) and MVM capsids (green). Colocalization areas appear yellow in the merge and are quantified by Image J analyzing 10 infected cells from three individual experiments. Scale bar 8 µm.
(PPTX)

Figure S2 **Induction of the parvoviral P38-promoter with a synthetic transactivator protein.** (A) Composition of the transactivator protein. The MVM NS1 DNA-binding domain to the TAR-element of the P38 promoter (NS1-DNA-B: aa 1–275) was fused to the acidic transactivator domain (NS1-TA: aa 545–672) spaced by the green fluorescent protein (GFP). The N-terminal glutathione-S-transferase (GST) serves to constitutively dimerize the polypeptide. (B) A9 cells grown on spot-slides were transduced with the indicated recombinant AAVs (rAAV:P4-Transactivator [TA], rAAV:P38-Myc-dnRab1 [dnRab1]) at 10^4^ genomes/cell. 72 h post transduction, the cells were fixed with paraformaldehyde, stained with Myc and LaminB antibodies and analyzed by confocal laser scanning microscopy for the presence of MycRab1. Scale bar: 30 µm. (C) Impact of rAAV:P38-dnRab1 transduction on GLuc secretion in the presence and absence of rAAV:P4-Transactivator. A9 cells were transfected with pCMV-GLuc and transduced with the indicated rAAVs at 10^4^ genomes/cell. The proportion of secreted GLuc in the medium was compared to control A9 cells. (D) Impact of Transactivator expression on cell metabolic activity. A9 or NCH149 cells grown on spot-slides were transduced (or not) with the indicated recombinant AAVs (rAAV:P4-Transactivator [TA], rAAV:P4-Myc-dnPDK1 [dnPDK1], rAAV:P4-NS1(MVM/H1) [PV-NS1]) at 10^4^ genomes/cell. 72 h post transduction, the cells were labeled for 30 min with Mitotracker. Mitochondrial activity was measured by confocal laser scanning microscopy, quantified with image J software, and expressed as relative light intensity per cell. Knockdown of the phosphoinositide-dependent kinase1, a key-regulator for cell metabolism by expression of dn PDK1 was used as a control for the downregulation of metabolic activity.
(PPTX)

Figure S3 **A9 cells grown on spot slides were infected (MVM 24 h) or not (A9) with MVMp (30 pfu/cell) and further incubated with B7 antibodies to neutralize progeny particles released into the medium.** When indicated, Rab-protein functioning was inhibited by over-expression of the dominant-negative Rab-variant (dnRab1, dnRab8, dnRab11), transduced by rAAV 24 h prior to parvovirus infection. Cells were fixed with paraformaldehyde 24 h p.i., and analyzed by confocal laser scanning microscopy after double-staining with MVM capsids (green) together with the cell proteins (red) Sec23 (ER) or Rab6 (golgi), respectively. Colocalization areas appear yellow in the merge and are quantified by Image J analyzing 10 infected cells from three individual experiments. Scale bar: 8 µm.
(PPTX)

Figure S4 **A9 cells grown on spot slides were infected (MVM 24 h) or not (A9) with MVMp (30 pfu/cell) and further incubated with B7 antibodies to neutralize progeny particles released into the medium.** When indicated, ERM-protein functioning was altered by over-expression of Rdx*dl*[P], RdxY146F, or MoeT547A, respectively, transduced by rAAV 24 h prior to parvovirus infection. Cells were fixed with paraformaldehyde 24 h p.i., and analyzed by confocal laser scanning microscopy after double-staining with MVM capsids (green) together with the cell proteins (red) Sec23 (ER), Rab6 (golgi), Rab8 (TGN→PM) or Rab11 (RE), respectively. Colocalization areas appear yellow in the merge and are quantified by Image J analyzing 10 infected cells from three individual experiments. Scale bar 8 µm.
(PPTX)

Figure S5 **Inhibition of second round infections by neuraminidase and neutralizing antibody treatment.** A9 cells were infected with MVMp (30 pfu/cell), harvested and fixed with paraformaldehyde at 24 h p.i. (Inf 1). Medium supernatants were then used for second round infections of naïve A9 cells in the presence/absence of serial dilutions of neutralizing antibodies (B7) or Neuraminidase, respectively (Inf 2). Second round infection rates were determined at 24 h p.i. by immunofluorescence microscopy detection of NS1 (αNS1) and capsid (αCap)-containing cells, after laminB counterstaining. Data shown were calculated using Image J from three independent experiments, each analyzing at least 200 cells.
(PPTX)

Figure S6 (**A**) **Transduction of target cell lines with rAAVs expressing effector genes under the control of the PV P4 or P38 promoter.** (A, B) A9 cells grown on spot-slides were transduced with rAAV-P4/P38-X (10^4^ genomes/cell) together with rAAV:P4-transactivator in the case of rAAV-P38-X. (A) At 72 h post transduction, the cells were fixed with paraformaldehyde and analyzed by confocal laser scanning microscopy for the presence of recombinant proteins. These proteins were detected using antibodies that recognize the corresponding N-terminal epitopes: Myc: for dnSar1(P38), dnRab1(P38), dnRab8(P38), dnRab11(P38), dnGln(P38); Flag: for MoeA(P4), Rdx*dl*[P](P4), RdxY(P4). Scale bar: 100 µm. (B) **Cytotoxicity of rAAV expressing effector constructs.** At 72 h post transduction, cells were either treated with mitotracker, fixed with paraformaldehyde and analyzed by confocal laser scanning microscopy, or treated with propidium iodide (PI) and observed by fluorescence microscopy. Three individual experiments each involving at least 200 cells were quantified using image J. For Mitotracker, fluorescence intensity per cell was determined and expressed relative to the negative control. For PI staining, positive cells were counted and % of lysis calculated. (C) **Antibody transfection efficiency and impact on virus infection.** A9 cells grown on spot-slides were transfected with control serum (αPKB) or neutralizing anti-Sec24 antibodies (αSec24) at 10 µg/5×10^5^cells. Twenty-four hours post-transfection, cells were infected with MVM, washed extensively after 2 h to remove the inoculum, further incubated for 24 h and fixed with paraformaldehyde. Transfected antibodies were detected with Alexa-conjugated secondary IgG and counterstained with DAPI to determine the proportion of transfected cells. The amount of infected cells was determined by means of NS1 expression. Scale bar 100 µm.
(PPTX)
